# Th17/1-Biased Inflammatory Environment Involved in the Response of Epithelial Cells to Antigen Stimuli in Nasal Polyps

**DOI:** 10.1155/2021/5531606

**Published:** 2021-06-09

**Authors:** Guangfu Xu, Silong Chen, Yuchun Dong, Li Xiao

**Affiliations:** ^1^Medical College of China Three Gorges University, China; ^2^The Department of Otolaryngology, ChangYang People's Hospital, China

## Abstract

Several studies showed that IL-17A was significantly increased in nasal polyps (NPs). However, the source and characteristics of IL-17A-producing cells in NPs were not fully understood. We isolated mononuclear cells from NPs and uncinate tissues and analyzed them using flow cytometry. The results indicated that IL-17A was increased in NP tissues compared to uncinate tissues. The main IL-17A-expressing cells were CD3^+^ T cells in NP tissues, including Th17 cells, Tc17 cells, and *γδ*T17 cells. Not similar to those in uncinate tissues, the majority of Th17 cells highly coexpressed IFN-*γ* in NP tissues, such as Th17/1 cells, which highly expressed CXCR3, CCR6, ROR*γ*t, and T-bet. Furthermore, Th17/1-biased environment increased the response of nasal epithelial cells to bacterial and viral stimuli, implying that Th17/1 cells play a greater role in the pathological development of NPs than Th17 or Th1 cells.

## 1. Introduction

Nasal polyps (NPs) are a chronic inflammatory disease of the upper airway with impaired quality of life which affects from 1% to 4% of the population [1]. Patients with NPs typically present with nasal obstruction, rhinorrhea or hyposmia, and so on. It is still one of the most challenging diseases in clinical rhinology because of its complex etiology and frequent recurrence [2]. Although the etiology and pathogenesis of NP have not been fully elucidated, it is probably a multifactorial disease with several different etiological factors in which bacteria, viruses, and fungi are closely related to the establishment of the inflammatory process [3]. Our earlier [4] and other studies [5] have demonstrated that different kinds of immune cells and a complicated cytokine response network were involved in the pathogenesis of immune-mediated disorders in NP tissues.

In recent years, the role of IL-17A in respiratory diseases has intensively been investigated. It was consensus that the expression of IL-17A was significantly upregulated in NP tissues [6] and significantly positively correlated with clinical features [7]. IL-17A may be involved in the development and remodeling of NPs through its local immune modulation [8]. However, the source and phenotype of IL-17A-producing cells in NP tissues are still not clear. In this study, we performed single-cell analysis to identify the IL-17A-producing cells and examined whether a Th17/1-biased cytokine environment regulated the response of nasal epithelial cells to viral and bacterial infections.

## 2. Materials and Methods

### 2.1. Patients

NP and uncinate tissues were recruited from ChangYang People's Hospital. 35 NP samples were obtained during routine functional endoscopic sinus surgery. The diagnosis was made according to the criteria of the European Position Paper on Rhinosinusitis and Nasal Polyps 2007 (EP3OS 2007) [9] and was based on patient history, clinical examination, nasal endoscopy, and sinus computed tomography (CT) scanning. For details of subjects' characteristics, see Table 1. 16 uncinate tissue samples were recruited from patients undergoing septoplasty during septal surgery. Patients with established immunodeficiency, pregnancy, diagnosis of classic allergic fungal sinusitis, or cystic fibrosis were excluded from the study. None of the subjects used oral or intranasal steroids for at least 2 weeks before sample collection. All subjects signed informed consent forms, and the study was approved by the local ethics committees of participating hospitals.

### 2.2. Cell Isolation and Tissue Homogenate

Tissue samples were cut into small pieces, digested in incomplete RPMI-1640 with endotoxin-free collagenase I (2 mg/ml, Sigma-Aldrich, St Louis, MO, USA) for 30 min at 37°C. The digested fragments were filtered through a 100 mm cell nylon mesh (BD Bioscience PharMingen, San Diego, CA, USA) to prepare a single-cell suspension. The mononuclear cells from NP and uncinate tissues were obtained by Ficoll-Hypaque (Tianjin Hao Yang Biological Manufacture, Tianjin, China) density gradient centrifugation.

Fresh tissue specimens were homogenized on ice for 3 min with PBS (1 ml of PBS/100 mg of tissue) containing a protease inhibitor cocktail (Keygentec, Nanjing, Jiangsu, China). The homogenates were then centrifuged at 4000 rpm for 20 minutes at 4°C, and the supernatants were stored at -80°C for ELISA.

### 2.3. Flow Cytometry

Cells were firstly incubated with red fluorescent reactive dye (live/dead fixable dead cell stain kit, Invitrogen) for 30 minutes for dead cell discrimination. Then, the cells were resuspended with PBS buffer containing 0.1% BSA and 0.05% sodium azide. For surface staining, cells were incubated with the respective mAbs at 4°C in the dark for 30 min. For the detection of intracellular cytokines, cells were fixed with 4% paraformaldehyde and permeabilized in PBS buffer containing 0.1% saponin (Sigma-Aldrich), 0.1% BSA, and 0.05% NaN_3_ for at least 2 h at 4°C and then stained with conjugated mAbs. For the detection of intracellular transcription factors, cells were stained for surface antigens, followed by fixation, permeabilization with Permeabilization/Fixation buffer (BD Bioscience PharMingen), and staining according to the protocol of the Permeabilization/Fixation Kit. Stained cells were washed twice before analysis using a BD FACS AriaII flow cytometer (San Jose, CA, USA). Lymphocytes were gated on forward and side scatter profiles and analyzed using FlowJo software (Treestar, San Carlos, CA, USA).

The following mAbs were used for cell surface or intracellular staining: FITC-labeled anti-CD45RO (clone: UCHL1), anti-CD69 (clone: FN50), PE-labeled anti-IL-17 (clone: N49-653), anti-T-bet (clone: 4B10), anti-CD25 (clone: 2A3), APC-Cy7-labeled anti-CD4 (clone: GK1.5), APC-labeled anti-CD62L (clone: DREG-56), anti-CCR7 (clone: 3D12), PE-CF594-labeled anti-CD3 (clone: SP34-2), isotype-matched control antibodies, purified anti-CD3, and anti-CD28 mAb which were purchased from BD Bioscience PharMingen. FITC-labeled anti-IL-17 (clone: eBio64DEC17), Pecy7-labeled anti-IFN-*γ* (clone: 4S.B3), Percp-Cy5.5-labeled anti-IL-4 (clone: 8D4-8), APC-labeled anti-IL-22 (clone: IL22JOP), and anti-ROR*γ*t (clone: AFKJS-9) were purchased from eBioscience (Santiago, Chile).

### 2.4. Cell Culture Conditions

To analyze the expression of cytokines and transcription factors, mononuclear cells from NP and uncinate tissues were stimulated with PMA (20 ng/ml; Sigma-Aldrich) and ionomycin (1 *μ*g/ml; Sigma-Aldrich) at 37°C with 5% CO_2_ in the presence of brefeldin A (10 *μ*g/ml; Sigma-Aldrich) for 5 h.

Human nasopharyngeal epithelial cell line NP69 was incubated in complete RPMI-1640 medium at 37°C and 5% CO_2_. To analyze the responses to virus/bacteria-like stimuli, NP69 cells were seeded in 6-well plates (2 × 10^5^ cells/well) in the presence or absence of human IFN-*γ* and IL-17A (both at 2 ng/ml, Gibco) for 48 hours. Then, the cells were stimulated with either poly I:C (10 *μ*g/ml; InvivoGen) or LPS (300 ng/ml; Sigma-Aldrich) for 4 h in serum-free media. Cells cultured in the medium were used as negative controls. Five independent experiments were performed.

### 2.5. ELISA

IL-17A (eBioscience), IFN-*γ*, IL-6, and IL-8 (BD Bioscience PharMingen) production was assayed according to the manufacturer's protocols. The detection limits were as follows: IL-17, 4 pg/ml; IFN-*γ*, 4.7 pg/ml; IL-6, 1.7 pg/ml; and IL-8, 1.82 pg/ml. For convenient analysis, all values of less than the detectable limit were considered zero.

### 2.6. ELISpot and Q-PCR

Mononuclear cells from NP and uncinate tissues were stimulated with anti-CD3 and anti-CD28 (both 1 *μ*g/ml, Sigma-Aldrich) for 24 h. Then, the frequencies of IL-17-producing cells were detected by ELISpot. Spot-forming cells (SFC) were enumerated with the ELISpot image analysis system (Champspot II, Sage Creation, Beijing, China).

The primers used in PCR are shown in Table 2.

### 2.7. Statistical Analysis

Data are expressed as the mean ± SEM or median (range). Comparison between two groups was performed by Student's *t*-test. Value of *p* < 0.05 (two-tailed) was considered significant.

## 3. Results

### 3.1. Increased IL-17A in NP Tissues

**Figure 1:**
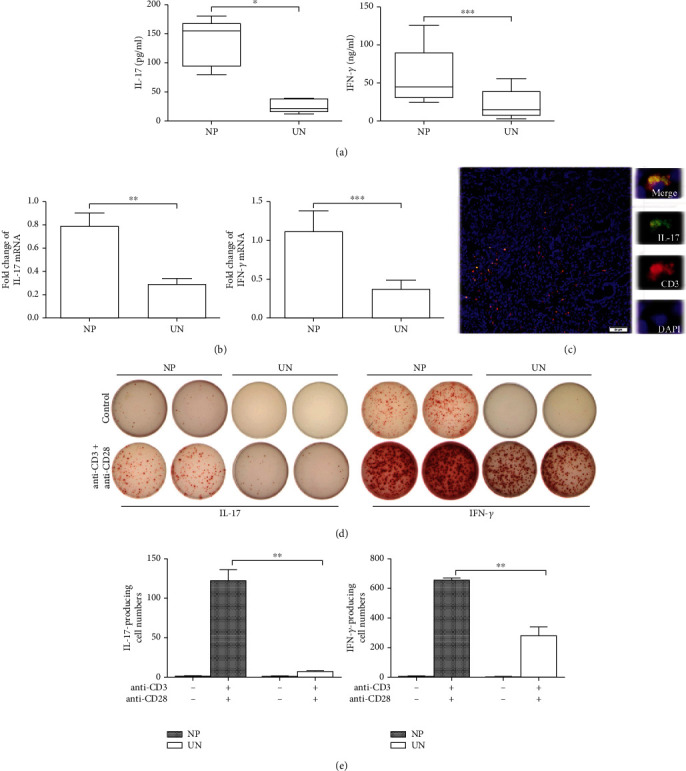
Increased IL-17A in NP tissues. (a) Summary data showed the concentrations of IL-17A and IFN-*γ* in the homogenates of NP (*n* = 18) and uncinate (*n* = 10) tissues. (b) Summary data showed mRNA expression of IL-17A and IFN-*γ* in NP (*n* = 18) and uncinate (*n* = 10) tissues. (c) Representative microphotographs of CD3 (red)-, IL-17 (green)-, and DAPI (blue)-stained section of NP tissues. Scale bars represent 50 mM. (d) The mononuclear cells from NP and uncinate tissues were cultured with or without anti-CD3 plus anti-CD28 for 24 h. Representative ELISpot results of IL-17A- and IFN-*γ*-producing cells were shown. (e) Summary ELISpot results of IL-17A- and IFN-*γ*-producing cells were shown (*n* = 5). Data were shown as the mean ± SEM. Statistical significance was determined with the Mann–Whitney test. ^∗^*p* < 0.05; ^∗∗^*p* < 0.01; ns: no significance; NP: nasal polyp; UN: uncinate.

We collected NP and uncinate samples and found that there were significantly increased protein and mRNA expressions of IL-17A and IFN-*γ* in NP tissues than in uncinate tissues (*p* < 0.01, Figures 1(a) and 1(b)). Immunofluorescence histochemical analysis revealed IL-17-producing CD3^+^ cells (Figure 1(c)). The ELISpot assay showed that enhanced IL-17A- and IFN-*γ*-producing cells in the mononuclear cells were induced from NP tissues than uncinate tissues in the addition of anti-CD3 and anti-CD28 (*p* < 0.05, Figures 1(d) and 1(e)). These data indicated that IL-17A was increased in NP tissues compared to uncinate tissues.

### 3.2. The Cell Sources of IL-17 Production in NP Tissues

**Figure 2:**
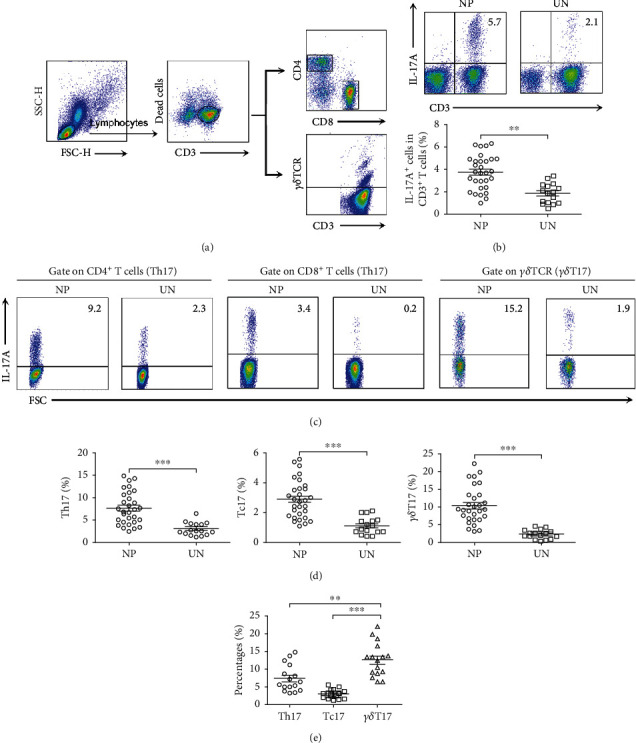
Both CD4^+^, CD8^+^, and *γδ*T cells expressed IL-17A in NP tissues. (a) FACS gating was used in the analysis of CD3^+^ T cells. (b) Representative FACS graphs and summary data showed the expression of IL-17A by CD3^+^ T cells in NP (*n* = 30) and uncinate (*n* = 16) tissues. (c, d) Representative FACS graphs and summary data showed the expression of IL-17A by CD4^+^, CD8^+^, and *γδ*T cells in NP (*n* = 30) and uncinate (*n* = 16) tissues. (e) Summary data showed the percentages of Th17, Tc17, and *γδ*T17 cells in NP tissues (*n* = 16). Data were shown as the mean ± SEM. Statistical significance was determined with the Mann–Whitney test. ^∗∗^*p* < 0.01; ^∗∗∗^*p* < 0.001. NP: nasal polyp; UN: uncinate.

To investigate the source of IL-17A, we performed a single-cell analysis by FACS using lymphocytes isolated from NP tissues and uncinate tissues. The results showed that CD3^+^ T cells were the major producing cells of IL-17A in NP tissues. And the fraction of IL-17A-expressing CD3^+^ T cells in NP tissues was significantly higher compared with uncinate tissues (*p* < 0.01, Figures 2(a) and 2(b)). Further study demonstrated that IL-17A was produced by CD4^+^ T cells (Th17), CD8^+^ T cells (Tc17), and *γδ*T cells (*γδ*T17) (Figure 2(c)). The percentages of Th17, Tc17, and *γδ*T17 cells were increased in NP tissues than in uncinate tissues (*p* < 0.01, Figure 2(d)). Among all IL-17A-producing CD3^+^ T cells, the percentages of *γδ*T17 cells were much more than Th17 and Tc17 cells in NP tissues, suggesting that *γδ*T cells were the most potent IL-17A-producing T cells (*p* < 0.01, Figure 2(e)).

### 3.3. The Majority of Th17 Cells Highly Coexpressed IFN-*γ* in NP Tissues

**Figure 3:**
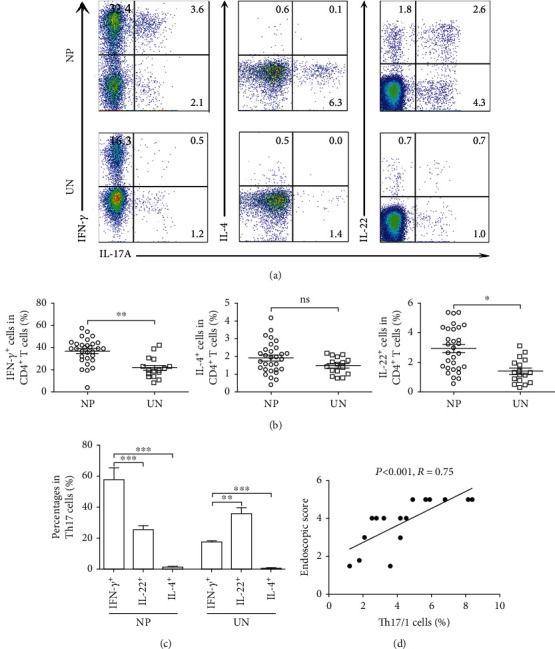
The majority of CD4^+^IL-17^+^ cells coexpressed IFN-*γ* cells in NP tissues. (a, b) Representative graph and summary data showed the expression of IL-17A, IFN-*γ*, IL-22, and IL-4 by Th17 cells in NP (*n* = 30) and uncinate (*n* = 16) tissues. (c) Summary data showed the expression of IFN-*γ*^+^, IL-22^+^, or IL-4^+^ cells in Th17 cells in NP (*n* = 30) and uncinate (*n* = 16) tissues. (d) The percentage of Th17/1 cells in NP tissues was correlated with endoscopic scores (*n* = 16). Correlations were determined by Spearman's rank correlation coefficients. Data were shown as the mean ± SEM. Statistical significance was determined with the Mann–Whitney test. ^∗^*p* < 0.05; ^∗∗^*p* < 0.01; ns: no significance; NP: nasal polyp; UN: uncinate.

Then, we determined whether IL-17A-producing cells coexpressed Th1- and Th2-related cytokines in NP tissues. FACS analysis indicated that CD4^+^IL-17^+^ T cells expressed higher percentages of IFN-*γ* and IL-22 in NP tissues than uncinate tissues; the expression of IL-4 by Th17 cells was not significantly different (Figures 3(a) and 3(b)). IL-17A-producing CD4^+^ T cells mainly coexpressed IL-22 in uncinate tissues. However, the majority of CD4^+^IL-17^+^ T cells coexpressed IFN-*γ* and to a lesser extent IL-22 in NP tissues (Figure 3(c)). These data suggested that most of IL-17A^+^CD4^+^ T cells were IFN-*γ*-producing cells in NP tissues, as Th17/1 cells. More importantly, the percentage of Th17/1 cells in NP tissues was positively related to endoscopic scores of NP patients (Figure 3(d)).

### 3.4. The Phenotype of Th17/1 Cells in NP Tissues

**Figure 4:**
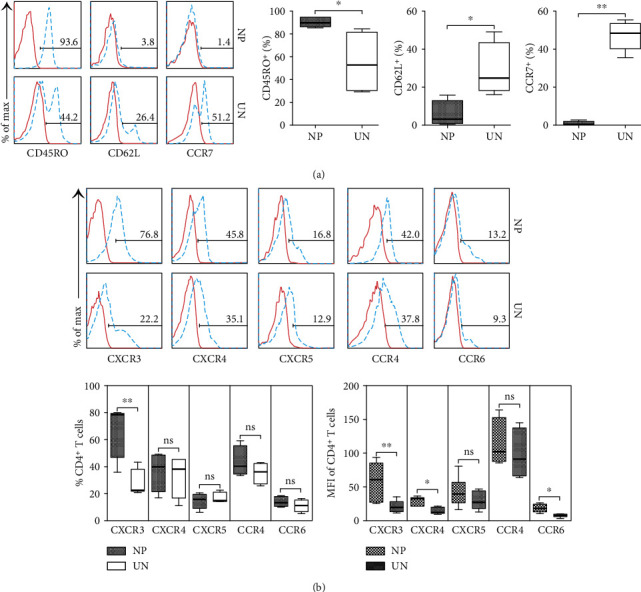
The phenotype of CD4^+^L-17^+^IFN-*γ*^+^ T cells from NP tissues. (a, b) Representative FACS graphs and summary data showed the expression of CD69 and CD25 on CD3^+^ T cells of NP (*n* = 12) and uncinate (*n* = 9) tissues. (c, d) Representative FACS graphs and summary data showed the expression of CD45RO, CD62L, and CCR7 on CD3^+^ T cells of NP (*n* = 12) and uncinate (*n* = 9) tissues. Data were shown as the mean ± SEM. Statistical significance was determined with the Mann–Whitney test. ^∗^*p* < 0.05; ^∗∗^*p* < 0.01; ns: no significance; NP: nasal polyp; UN: uncinate.

We analyzed the basic phenotype of CD4^+^IL-17^**+**^IFN-*γ*^+^ T cells from NP tissues. The results showed that the majority of CD4^+^IL-17^**+**^IFN-*γ*^+^ T cells highly expressed CD45RO, but less of CD62L and CCR7 in NP tissues (*p* < 0.05, Figure 4(a)), displaying an effector memory phenotype. And more, CD4^+^IL-17^**+**^IFN-*γ*^+^ T cells expressed higher levels of chemokine receptors CXCR3, CXCR4, and CCR6 in NP tissues than in uncinate tissues (*p* < 0.05, Figure 4(b)).

**Figure 5:**
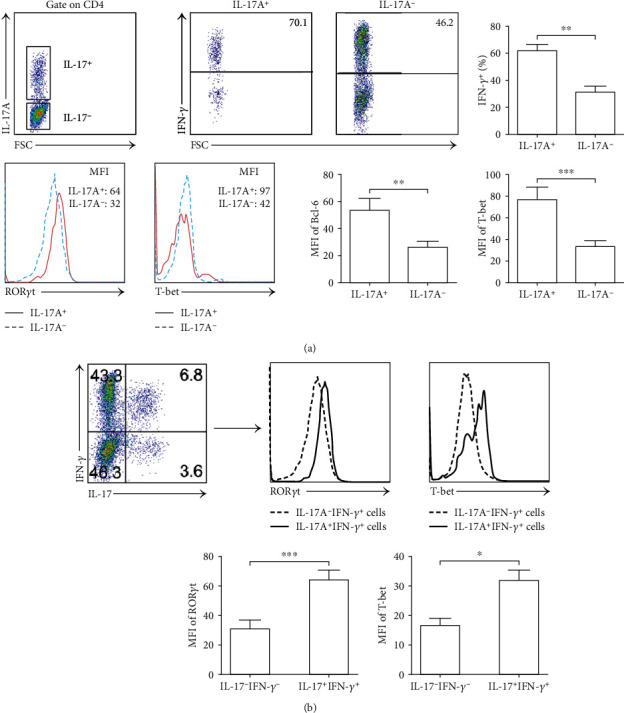
Transcription factor expression in CD4^+^L-17^+^IFN-*γ*^+^ T cells from NP tissues. (a) IL-17A^+^ and IL-17A^−^ cells were gated on CD4^+^ T cells in NP tissues. The expression of IFN-*γ*, ROR*γ*t, and T-bet was analyzed (*n* = 12). (b) CD4^+^ T cells from NP tissues were gated on the expression of IL-17A and IFN-*γ*. Representative FACS and summary data showed the expression of ROR*γ*t, T-bet in IL-17^+^IFN-*γ*^+^, and IL-17^−^IFN-*γ*^−^ cells (*n* = 12). Data were shown as the mean ± SEM. Statistical significance was determined with the Mann–Whitney test. ^∗^*p* < 0.05; ^∗∗^*p* < 0.01; ^∗∗∗^*p* < 0.001.

Because of the contribution of transcription factors to cytokine expression, we analyzed the expression of ROR*γ*t (for IL-17) and T-bet (for IFN-*γ*) by Th17 cells. We observed that IL-17A^+^ cells expressed significantly higher amounts of IFN-*γ*, ROR*γ*t, and T-bet than did IL-17A^−^ cells in NP tissues (*p* < 0.01, Figure 5(a)). To further evaluate the regulation of transcription factors, the CD4^+^ T cells in NP tissues were gated according to the expression of IL-17A and IFN-*γ*. The expression of ROR*γ*t and T-bet was analyzed in each cell subpopulation. Compared to IL-17A^−^IFN-*γ*^−^ T cells, IL-17A^+^IFN-*γ*^+^ T cells highly expressed ROR*γ*t and T-bet (*p* < 0.05, Figure 5(b)). These results demonstrated that Th17/1 in NP tissues not only secreted multiple cytokines but also expressed many transcription factors that should be considered integral to regulating the differentiation and function of nasal polyp-infiltrating lymphocytes.

### 3.5. The Response of Epithelial Cells to Stimuli in a Th17/1-Biased Environment

**Figure 6:**
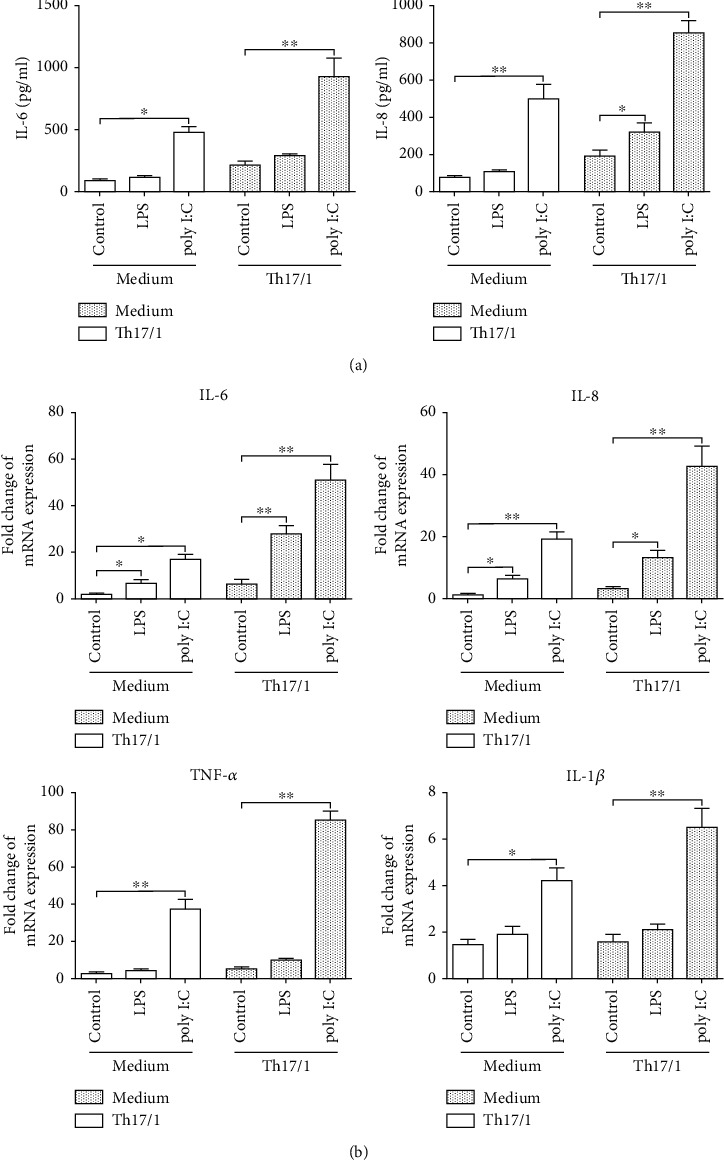
Effect of a Th17/1 environment on the proinflammatory response of NP69 cell to stimuli. NP69 cells were cultured in the presence or absence of human IFN-*γ* and IL-17A for 48 hours, then stimulated with either poly I:C or LPS for 4 h in serum-free media. (a) Summary data showed IL-6 and IL-8 expression levels by NP69 cells (*n* = 5). (b) Summary data showed mRNA expression of IL-6, IL-8, TNF-*α*, and IL-1*β* in NP69 cells (*n* = 5). Data were shown as the mean ± SEM. Statistical significance was determined with the Mann–Whitney test. ^∗^*p* < 0.05; ^∗∗^*p* < 0.01; ^∗∗∗^*p* < 0.001.

To investigate the effects of Th17/1 cytokines, human nasopharyngeal epithelial cell NP69 was cultured in the presence of the major Th1 cytokine IFN-*γ* and Th17 cytokine IL-17A. Then, the responses of cells to stimulation with poly I:C or bacterial LPS were analyzed. NP69 cell expression of mRNA for IL-6, IL-8, TNF-*α*, and IL-1*β* in response to poly I:C was significantly upregulated in Th1/17 cytokine condition when compared to media alone. And poly I:C was the most potent stimulus for increasing the expression of proinflammatory factors. In the meantime, IL-6 and IL-8 responses to LPS were also enhanced in a Th1/17 environment, relative to media alone (Figure 6). These data showed that Th17/1-biased environment increased the response of nasal epithelial cells to bacterial and viral-like stimuli.

## 4. Discussion

IL-17A is a proinflammatory cytokine that plays an essential role in host defense against microbial infections and is implicated in various inflammatory conditions. Pathological production of IL-17A leads to excessive inflammatory response and overt tissue damage [10]. Previous reports have showed an enhanced IL-17A expression regardless of eosinophilic or noneosinophilic inflammation of CRSwNP [11], despite different results about the role of IL-17A in the severity and development of NPs in the different regions [12, 13]. The significance of IL-17A in NPs of the Asia area may be more important than that in Europe. Recent studies have shown that IL-17A induced recruitment not only of neutrophils [14] but also of eosinophils in NPs [15]; increased IL-6, IL-11, IL-9, and G-CSF production in human bronchial fibroblasts; and promoted remodeling [16]. In this study, our results also demonstrated that IL-17A was significantly increased in NP tissues than in uncinate tissues. In the upper airway [17], the cellular sources for IL-17A are reported to be T lymphocytes, neutrophils, eosinophils, and epithelial cells. However, the cellular sources of IL-17A are not well understood. We further found that IL-17A was almost exclusively secreted by CD3^+^ T cells including Th17, Tc17, and *γδ*T17 cells in NP tissues, in which Th17 cells were the major source of IL-17A. Moreover, *γδ*T cells were the most potent IL-17A-producing cells in NP tissues, though the proportion of *γδ*T cells was less than that of CD4^+^ T and CD8^+^ T cells.

NPs are a common nasal inflammatory disease with regional differences. The pathological features of NPs are characterized by the infiltration of multiple types of inflammatory cells and mixed patterns of T cell response [18]. According to the European position paper on rhinosinusitis and nasal polyps 2020, it is generally recognized that chronic rhinosinusitis with nasal polyps (CRSwNP) is characterized by infiltration of eosinophils and excessive expression of Th2 cytokines [19]. And recent reports have demonstrated that Chinese or Korean CRSwNP patients possess a distinct pathogenic phenotype including the accumulation of neutrophils and mixed Th1/Th17 response [20, 21]. Many studies reported that Th17 cells expressed both IL-17A and IFN-*γ* at the same time in some of the pathological conditions, indicating a high degree of developmental flexibility [22]. The developmental flexibility of Th17 cells and their shift to a Th1 cell-like phenotype has been linked to the pathogenicity of Th17 cells in infectious diseases [23], autoimmune diseases [24], and tumors [25]. We analyzed the cytokine expression of Th17 cells in NP tissues using flow cytometry. The results showed that the majority of IL-17A-expressing CD4^+^ T cells coexpressed IFN-*γ*, not IL-4 or IL-22, and there was a significant increase of Th17/1 cells in NP tissues.

Published studies [26] have described that CD4^+^ T cells simultaneously produced IL-17A, and IFN-*γ* stably coexpressed Th17 and Th1 cell master transcription factors ROR*γ*t and T-bet. However, the coexpression of ROR*γ*t and T-bet was not sufficient to generate Th cells with dual Th17 and Th1 cell features. The development of IL-17A^+^IFN-*γ*^+^ or Th1-like Th17 cells was dependent on T-bet, Runx1, and Runx3 [27]. Our results demonstrated that CD4^+^IL-17A^+^IFN-*γ*^+^ T cell highly expressed CXCR3 and CCR6, T-bet, and ROR*γ*t in NP tissues. However, the precise function of IL-17A^+^IFN-*γ*^+^ cells in NP tissues needs further study.

In humans, IFN-*γ*-producing Th17 cells (Th17/1 cells), identified by coexpression of the chemokine receptors CCR6 and CXCR3, have been proposed to be highly pathogenic functions due in part to their expression of the proinflammatory cytokines IL-17, IFN-*γ*, and GM-CSF [28]. Reduction of Th1-like Th17 cell numbers is a potential strategy for the treatment of autoimmune diseases [29]. The acquisition of Th1 effector cell functions by Th17 cells, including IFN-*γ* expression, is crucial for the effective antitumor activity of Th17 cells [30]. However, their developmental requirements, relationship with classical Th17 and Th1 cells, and physiological role in normal immune responses are not well understood. We found that Th17/1-biased environment increased the response of nasal epithelial cells to bacterial and viral stimuli, implying that Th1-like Th17 cells play a greater role in the pathological development of NPs than Th17 or Th1 cells.

## Figures and Tables

**Table 1:** Subjects' characteristics.

Characteristic	NP
Subject numbers	35
Age (y), mean (range)	53 (43~72)
Sex (male/female)	23/12
Duration (y), mean (range)	12.5 (5–40)
Atopic	8
Aspirin intolerance	0
CT score	15.5 (6~22)
Nasal endoscopy score	4.5 (1~6)
Mean VAS score	5.4 (3~8.4)

**Table 2:** Sequence of primers used for qRT-PCR.

	Forward primer (5′-3′)	Reverse primer (5′-3′)	Product size (bp)
IL-17	AGATTACTACAACCGATCCACCT	GGGGACAGAGTTCATGTGGTA	151
IFN-*γ*	TCGGTAACTGACTTGAATGTCCA	TCGCTTCCCTGTTTTAGCTGC	93
IL-6	ACTCACCTCTTCAGAACGAATTG	CCATCTTTGGAAGGTTCAGGTTG	149
IL-8	TTTTGCCAAGGAGTGCTAAAGA	AACCCTCTGCACCCAGTTTTC	194
TNF-*α*	CCTCTCTCTAATCAGCCCTCTG	GAGGACCTGGGAGTAGATGAG	220
IL-1*β*	ATGATGGCTTATTACAGTGGCAA	GTCGGAGATTCGTAGCTGGA	132
GAPDH	TCAAGAAGGTGGTGAAGCAGG	TCAAAGGTGGAGGAGTGGGT	115

## Data Availability

The data that support the findings of this study are available from the corresponding author upon reasonable request.
